# Dosimetric comparison of organs at risk in ultra-hypofractionated versus hypofractionated postoperative radiotherapy for early breast cancer: single center clinical study

**DOI:** 10.2478/raon-2026-0008

**Published:** 2026-02-06

**Authors:** Gordana Petkovska, Ivica Ratosa, Valentina Bojovska Trajanovska, Marina Iljovska, Albina Pupakovski Creslovnik, Emilija Lazareva

**Affiliations:** University Clinic for Radiotherapy and Oncology,, Skopje, R. North Macedonia; Faculty of Medicine, University St. Cyril and Methodius, Skopje, R. North Macedonia; Division of Radiotherapy, Institute of Oncology Ljubljana, Ljubljana, Slovenia; Faculty of Medicine, University of Ljubljana, Ljubljana, Slovenia

**Keywords:** breast cancer, radiotherapy, ultra-hypofractionation, organs at risk

## Abstract

**Background:**

Growing evidence of safety and feasibility has prompted a shift toward ultra-hypofractionated (UHF) schedules in postoperative radiotherapy in early breast cancer.

**Patients and methods:**

Eighty patients over 50 years of age with early breast cancer (T1-2 and N0-1) who underwent postoperative, 3D conformal, free-breathing whole breast radiotherapy were included. The prospective arm consisted of 40 patients treated with UHF (26 Gy/5 fractions/one week) from 2023-2024, whereas the control arm was retrospective and represented by data from 40 patients treated with hypofractionated radiotherapy (HF) (40.5–42.2Gy/15–16 fractions/3 weeks) between 2015 and 2020. Dosimetric parameters for organs at risk (OARs) (heart and ipsilateral lung) were derived from the dose-volume histograms. Statistical evaluation was done with paired sample t-test and Mann-Whitney U test.

**Results:**

Dosimetric analysis revealed that patients treated with UHF schedule received significantly lower equivalent doses in 2 Gy fractions (EQD_2_Gy) to OARs compared with those treated with the HF schedule. The mean ipsilateral lung EQD_2_Gy dose was significantly lower in the UHF group (3.94 ± 2.1 Gy) than in the HF group (6.24 ± 2.4 Gy; *p* < 0.01). Among patients with left-sided breast cancer, the mean heart EQD_2_Gy dose was also significantly reduced in the UHF group (1.34 ± 0.5 Gy) compared with the HF group (3.02 ± 1.4 Gy; *p* < 0.01).

**Conclusions:**

These findings indicate a consistent dosimetric advantage of the UHF schedule, particularly in reducing radiation exposure to the heart and ipsilateral lung. These results support the dosimetric safety and feasibility of UHF schedules in early breast cancer treatment.

## Introduction

Breast cancer is the most common malignant disease in women with incidence of 11.7% or 2.3 million new cases per year. One of 8 women will be diagnosed with this malignant disease. In 2020, the mortality rate was 6.9%, making breast cancer the fourth most common cause of death among malignant diseases. By 2040, these figures are expected to increase by 40% to 50% in countries with low to middle incomes.^1,2,3^

Historically, radiotherapy as a breast cancer treatment started shortly after radium radioactivity discovery in 1898, but at that time there was no proof of its efficiency. The National Surgical Adjuvant Breast and Bowel Project (NSABP) B04 clinical study, comparing radical mastectomy, total mastectomy, and total mastectomy followed by irradiation, revolutionized breast cancer surgery and subsequently established irradiation as one of the most essential therapy modalities.^4^ The NSABP B-06 trial compared lumpectomy and axillary node dissection with or without breast radiation to modified radical mastectomy.^5^ In 1976, it demonstrated a significant difference in ipsilateral relapse rates between the lumpectomy group with radiotherapy (14.3%) and the lumpectomy group without radiotherapy (39.2%); however, there were no significant differences in overall survival (OS), disease-free survival (DFS), or distant DFS when compared to the total mastectomy group. These results from the NSABP group, together with the results from Milan group clinical study at the same time, led by Veronesi, evaluating quadrantectomy plus radiotherapy established breast conserving surgery plus radiotherapy as the preferred method of local treatment for patients with operable breast cancer.^6^

The clinical trials listed above, like every other trial conducted prior to 2003, employed traditional fractionation with 2 Gy per fraction. In post-operative radiotherapy treatment for breast cancer, scientists have shifted to hypofractionated regimens: moderate fractionation (2–5 Gy per fraction) and ultra-hypofractionation (> 5 Gy per fraction) due to advancements in tumor radiobiology and modern radiotherapy techniques. Breast cancer exhibits a low *a/β* ratio of approximately 4, akin to late-responding normal tissues, indicating that the sensitivity of breast cancer tissue to dose fractionation is equivalent to that of normal tissue; thus, hypofractionated (HF) radiation regimens appear to be appropriate.^7,8^ START A and B were two randomized clinical trials comparing conventional and HF postoperative radiotherapy in breast cancer.^9^ START A compared two fractionation schedules of 50 Gy/25 fractions (fx) and 41.6–39 Gy/13 fx.^10^ After 9.3 years of follow up, 139 locoregional relapses occurred without significant difference between the two groups (6.3%, *vs*. 7.4%, p = 0.65). In a START B clinical study, conventional fractionation (50 Gy/25 fx) was compared with HF schedule (40 Gy/15 fx).^11^ At 10 years, the rates of locoregional relapse were 4.3% for the 40 Gy arm and 5.5% for the 50 Gy arm, with no significant difference observed (p = 0.21). Importantly, late adverse effects such as breast induration, telangiectasias, or breast edema were markedly less prevalent in the hypofractionation group in both studies. These robust findings established HF postoperative radiotherapy as the standard of care in breast cancer treatment, irrespective of disease stage, and led to its endorsement in national and international clinical guidelines.^12,13,14^

The period of ultra-hypofractionated (UHF) clinical trials in breast cancer commenced with the UK FAST clinical study^15^, conducted from 2004 to 2007, in which patients were randomized between three radiotherapy schedules (50 Gy/25 fx/5 weeks, 30 Gy/5 fx/5 weeks and 28.5 Gy/5 fx/5 weeks). Acute skin adverse effects of ≥grade 3 were the highest in the conventional fractionated group at 10.9%, compared to 2.7% in the 30 Gy group and 1.9% in the 28.5 Gy group. This study was not designed to determine ipsilateral tumor relapse (IBTR) between arms, however a low incidence of 1.2% was observed at the 10-year follow-up. After long-term follow-up, both UHF arms demonstrated equivalent tumor control with acceptable late normal tissue toxicity, although the 28.5 Gy schedule showed better normal tissue tolerability compared to the 30 Gy arm. These results supported the potential of UHF in selected patient populations.^16^

FAST-Forward^17^ is a prospective clinical study, with pragmatic design that involved more than 4000 patients comparing HF postoperative radiotherapy (40 Gy/15 fx) with UHF of 26 Gy/5 fx or 27 Gy/5 fx. Patients older than 18 years, with tumors up to 5 cm, N0–N1, post-breast-conserving surgery or mastectomy (majority underwent breast conserving surgery) with axillary surgery as indicated, were initially included. In 2013 an amendment was made to exclude patients with favorable characteristics (tumors < 2 cm, patients over 65 years, grade 1 and 2, luminal A subtypes) and the trial focused on patients with higher risk. This change aimed to reduce variability in outcomes and enhance the statistical power to detect differences between treatment arms, focusing more precise on evaluation of the primary endpoint - IBTR. After a median of 71.5 months of follow-up, the first data showed that IBTR was 2.1% in the 40 Gy group, 1.7% in the 27 Gy group, and 1.4% in the 26 Gy group. Non-inferiority for UHF regimens was confirmed. Furthermore, the clinical evaluation of normal tissue effects indicated a markedly elevated risk of any moderate or severe effect in the breast or chest wall for the 27 Gy group compared to the 40 Gy group (odds ratio [OR] 1.55 [95% CI 1.32 to 1.83], p < 0.0001), with no significant difference observed between the 26 Gy and 40 Gy groups (1.12 [0.94 to 1.34], p = 0.20).The findings from the 10-year follow-up were reported at ESTRO 2025, indicating an IBTR of 3.6% for the control group (40 Gy), 3.0% for the 27 Gy group, and 2.0% for the 26 Gy group, thereby corroborating prior results. The hazard ratio (26 Gy *vs*. 40 Gy) was 0.62 which indicates non-inferiority and a trend toward superiority of 26 Gy.^18^ Clinician and patient reported late normal tissue effects confirmed previous data that 26 Gy is comparable to 40 Gy, but 27 Gy fractionation schedule has higher rate of late side effects, such as firmness, shrinkage and induration on the irradiated breast.^18^

Adaptation on UHF in breast cancer radiotherapy is getting more interest every day worldwide. The experience in this fractionation schedule is getting richer and more real-world data is being published. Shortened treatment schedule enhances patient comfort in the postoperative setting without compromising treatment safety or therapeutic efficacy. Motivated by the promising 5-year outcomes of the FAST-Forward trial, we initiated this clinical study to evaluate the feasibility of UHF radiotherapy in patients with early-stage breast cancer. The primary objective of the study was to determine whether UHF could reduce exposure to surrounding normal tissues.

## Patients and materials

This study employs a combination of prospective and retrospective design. Before starting the research, interventional protocol was approved by Institutional review board and Ethical committee at Faculty of Medicine, Skopje (formal reference number is not issued by this authority). The study was conducted at the University clinic for radiotherapy and oncology, Skopje, Republic North Macedonia.

### Patients and treatment planning

In the prospective phase of the study (the intervention group) 40 patients in the period between January 2023 and August 2024 were included. Including criteria comprised: women aged ≥50 years, breast conserving surgery and defined nodal status (axillary dissection, sentinel node dissection or sentinel node dissection and consecutive axillary dissection), invasive or in situ breast cancer, complete tumour resection (R0), tumour size ≤ 5 cm (T1–2), 0–3 positive lymph nodes (N0–N1) and indication for radiotherapy. Patients with recurrent or metastatic breast cancer, other malignant diseases, contralateral breast cancer diagnosed within five years, or those who underwent breast reconstruction were excluded from the study. 3D conformal (3D-CRT) post-operative whole breast radiotherapy (WBRT) in this group was delivered in ultra-hypofractionation schedule of 26 Gy/5 fx/1 week. The control group comprised retrospective data from institutional medical records of 40 patients, treated in the period between 2015 and 2020, who fulfilled the identical inclusion and exclusion criteria as the intervention group, and who received 3D-CRT WBRT in a HF schedule of 40.5–42.4 Gy administered in 15–16 fractions over 3 weeks. A free-breathing, non-enhanced simulated computed tomography (CT) scan was performed on each patient. All women received treatment in the supine position, with both arms over the head, on an inclined simulation table with a breast board. The treatment planning and contouring processes were identical for patients in both groups and conducted using Varian EclipseTM software. Target volumes and organs-at-risk (OARs) were delineated according to Radiation Therapy Oncology Group (RTOG) atlas and three-dimensional radiotherapy (3D-CRT) plans were carried out according to institutional protocols.

Planning target volume for evaluation (PTVeval) was described as a structure 5 mm under the chest skin, subtracted from PTV. Target volumes were optimized as follows: PTVeval_95% > 95% (the volume of PTVeval receiving 95% of the prescribed dose as a percentage), PTVeval_max% ≤ 110%, PTVeval_105% ≤ 5 cm^3^, and PTVeval_107% ≤ 2 cm^3^ (the volumes receiving 105% and 107% of the prescribed dose expressed as percentages, respectively). The parameters assessed from OARs included: mean ipsilateral lung dose (Lung_mean), volume of the ipsilateral lung that receives 8 Gy expressed in % (LungV8 [%] for UHF and volume of the ipsilateral lung that receives 18 Gy expressed in % (LungV18 [%]) for HF, mean heart dose (Heart_ Dmean), volume of heart expressed in % that receives 1.5 Gy (HeartV1.5 [%] and volume of the heart expressed in % that receives 20 Gy (HeartV20 [%]) for the UHF and HF groups, respectively. Data was obtained from the dose-volume histograms for patients in both groups. Equivalent dose in 2 Gy fractions (EQD2) was calculated for all treatment plans using the formula:

$$EQD2=Dx\frac{d+\alpha /\beta }{2+\alpha /\beta }$$

(D = total dose, d = dose per fraction, *α/β =* alpha/beta ratio for tissue sensitivity (Gy)), with an α/β of 4 for the heart and 3 for the lung.

### Statistical analysis

The statistical analysis was conducted using the *Statistical Package for the Social Sciences* (SPSS, version 25.0; IBM Corp., Armonk, NY, USA). Data distribution was assessed with the Kolmogorov–Smirnov test and Shapiro–Wilk test. Quantitative variables are presented as arithmetic mean with standard deviation or as median values, while qualitative variables are presented as absolute and relative frequencies. Bivariate analyses were performed to compare the two radiation techniques. For qualitative features, the Pearson chi-square test and Fisher’s exact test were applied. For quantitative features, the Student’s *t*-test and Mann–Whitney *U* test were used. A *p*-value of < 0.05 was considered statistically significant.

## Results

The study included 80 patients aged between 50 and 75 years old with a mean age of 61.1 ± 6.6 years. Thirty-eight patients (45%) had right-sided breast cancer, and 42 (52.5%) patients had leftsided breast cancer. Chemotherapy was administered in 28 (35%) of the patients, targeted therapy in 13 (16.3%), and hormonal therapy in 70 (87.5%). According to tumor characteristics, the most prevalent histology was invasive breast cancer of no particular type (NST) in 56 (70%) of the patients. The average tumor size was 18.1 ± 8.2 mm (range 1-45 mm), and the most common tumor stage was T1c in 37 (46.3%) patients. Positive lymph nodes have been observed in 5 patients (6.3%). More than half of the patients had a stage of I breast cancer (47.8%), moderately differentiated tumors (51.8%), hormone receptor positive/HER2 negative breast cancer (59.8%), and progesterone levels greater than 10% (50.6%). Lymphovascular invasion was detected in 42 cases (52.5%).

As seen in Tables 1 and 2, the distribution of patient and tumor characteristics was comparable between the two groups. No statistical significance was found according to patients age, tumor side, adjuvant treatment with chemotherapy, hormonal therapy, targeted therapy, breast size, T stage, N stage, lymphovascular invasion, grade, histopathological or immunohistochemical type. The sole statistical difference seen pertained to tumor size, with the UHF group exhibiting tumors that were, on average, 4 mm larger than those in the HF group (p = 0.037).

**TABLE 1.** Patients’ characteristics

Variable	n	UHF	HF	p-level
**Age (years)**		**mean ± SD**	**min-max**	
UHF	40	60.8 ± 7.1	50–75	t = 0.37
HF	40	61.35± 6.2	50–74	p = 0.712
**Side**				
Right	38	22 (55)	16 (40)	X^2^ = 1.8
Center	42	18 (45)	24 (60)	p = 0.18
**Chemotherapy**				
No	50	24 (63.2)	26 (65)	X^2^ = 0.029
Yes	28	14 (36.8)	14 (35)	p = 0.865
**Hormonal therapy**				
No	8	2 (5.3)	6 (15)	X^2^ = 1.09
Yes	70	36 (94.7)	34 (85)	p = 0.297
**Target therapy**				
No	65	32 (84.2)	33 (82.5)	X^2^ = 0.041
Yes	13	6 (15.8)	7 (17.5)	p = 0.839
**Breast size**				
< 1300 cm^3^	68	36 (90)	32 (80)	X^2^ = 1.57
> 1300 cm^3^	12	4 (10)	8 (20)	p = 0.21

1 HF = hypofractionated group; n = number; UHF = ultra-hypofractionated group

**TABLE 2.** Tumor characteristics

Variable	n	UHF	HF	p-level
**Tumor stage**				
1b	7	2 (5.26)	5 (12.5)	p = 0.185
1c	37	18 (47.37)	19 (47.5)	
1mi	3	0	3 (7.5)	
2	31	18 (47.37)	13 (32.5)	
all	78	38	40	
**Tumor size**				
mean ± SD		20.3 ± 8.2	16.3 ± 7.9	t = 2.13 *p = 0.037
min-max		7–45	1–35	
median (IQR)		19 (15–22)	15 (11.5–23)	
**Lymph nodes**				
N0	73	36 (94.74)	37 (92.5)	X^2^ = 0.0035 p = 0.953
N1	5	2 (5.26)	3 (7.5)	
**Grade**				
G1	10	3 (8.11)	7 (17.5)	X^2^ = 191 p = 0.384
G2	51	27 (72.97)	24 (60)	
G3	16	7 (18.92)	9 (22.5)	
**Lymphovascular invasion**				
LV 0	42	21 (61.76)	21 (56.76)	X^2^ = 0.18 p = 0.668
LV 1	29	13 (38.24)	16 (43.24)	
**Histopathological type**				
NST	56	29 (72.5)	27 (67.5)	p = 0.23
lobular	11	6 (15)	5 (12.5)	
mixed	4	2 (5)	2 (5)	
other	7	1 (2.5)	6 (15)	
Tis	2	2 (5)	0	
**ER/HER2**				
ER+ HER2+	8	4 (10.53)	4 (10)	p = 0.344
ER+ HER2-	59	31 (81.58)	28 (70)	
ER-HER2+	3	0	3 (7.5)	
ER-HER2-	8	3 (7.89)	5 (12.5)	

1 ER = estrogen receptor; G = Grade; HER2 = human epidermal growth factor receptor 2; HF-RT = hypofractionated group; IQR = interquartile range; LV = lymphovascular invasion; N = Node; NST = non specific type; SD = standard deviation; Tis = tumor in situ; UHF-RT = ultra-hypofractionated group

All patients were treated with 3D-CRT. However, the irradiation periods differed between the study arms, with the intervention arm treated in 2023–2024 and the control arm in 2015–2020. Dosimetric results for target volume PTVeval and OARs (heart and lung), together with the statistical comparison between the two study arms are summarized in Tables 3, 4 and 5.

**TABLE 3.** Dosimetric evaluation of PTV

	PTV95%			p-level
	n	mean ± SD	min–max	
**UHF**	40	99.3 ± 0.6	97.4 – 100	t = 4.9 ***p = 0.000006
**HF**	40	98.3 ± 1.2	95.2 – 99.9	

1 HF = hypofractionated group; n = number; PTV = planning target volume; UHF = ultra-hypofractionated group

**TABLE 4.** Dosimetric evaluation of dose to the Heart

	HeartV(%))				p-level
	n	mean ± SD	min–max	median (IQR)	
**UHF**	19	7.7 ±4.0	0–16.3	7.5 (5.7–9.5)	Z = 3.3 **p = 0.0011
**HF**	24	3.7 ± 2.9	0.03–10.0	3.1 (1.4–5.0)	

	Heart_mean (Gy)				p-level
	n	mean ± SD	min–max	median (IQR)	
**UHF**	19	0.8 ± 0.3	0.5–1.54	0.7 (0.6–1.01)	Z = 5.2 p < 0.0001
**HF**	25	2.6 ± 1.3	0.9–5.4	2.4 (1.8–3.4)	

	Heart mean EQD2Gy α/β (4)			p-level
	n	mean ± SD	min–max	
**UHF**	19	1.3 ± 0.5	0.8–2.5	t = 4.9 ***p = 0.000017
**HF**	25	3.02 ± 1.4	1.1–6.1	

1 EQD2 = equivalent dose in 2 Gy fraction; HeartV1.5 (%) = volume of heart expressed in % that receives 1.5 Gy; HeartV20 (%) = volume of the heart expressed in % that receives 20 Gy; HF = hypofractionated group; IQR, interquartile range; UHF = ultra-hypofractionated group

**TABLE 5.** Dosimetric evaluation to the Lung

	LungVGy(%)				p-level
	n	mean ± SD	min–max	median (IQR)	
**UHF**	40	8.1 ± 4.1	0.2–15.5	7.95 (5.5–11.1)	Z = 2.59 **p = 0.0096
**HF**	40	11.8 ± 5.6	1.6–24.5	10.25 (7.5–16.2)	

	Lung_mean(Gy)				p-level
	n	mean ± SD	min–max	median (IQR)	
**UHF**	40	2.5 ± 1.3	0.8–8.5	2.4 (1.8–3.1)	Z = 6.51 p < 0.0001
**HF**	40	5.6 ± 2.1	1.8–10.2	5.135 (3.9–7.3)	

	Lung mean EQD2Gy a/β (4)				p-level
	n	mean ± SD	min–max	median (IQR)	
**UHF**	40	3.9 ± 2.1	1.2–13.9	3.64 (2.8–4.8)	Z = 4.76 ***p = 0.000002
**HF**	40	6.2 ± 2.4	2.1–11.3	5.715 (4.4–8.1)	

1 EQD2 = equivalent dose in 2Gy fraction; HF = hypofractionated group; IQR = interquartile range; LungV8(%) = volume of ipsilateral lung expressed in % that receives 8 Gy; LungV18 (%) = volume of ipsilateral lung expressed in % that receives 18Gy; UHF = ultra-hypofractionated group

## Discussion

This study aimed to assess the dose received by the organs at risk (heart and ipsilateral lung) among patients treated with two distinct fractionation schedules: UHF and HF. Statistical analysis demonstrated that patients in the UHF group consistently received significantly lower doses to both the heart and lung compared with those in the HF group, highlighting the dosimetric advantage of this regimen.

Most of the published studies about UHF in breast cancer are analyzing the impact on this schedule on acute skin toxicity and/or local control of the disease. Even in FAST Forward study^17^ dosimetric assessment on the OARs was not published. There is data about contralateral breast cancer and death from other causes (second cancer, cardiac death) that cannot be corelated directly.^17^

In 2022, Ivanov *et al*.^19^ published a study that found considerably lower doses in OARs in the patients treated with UHF, similar to findings in our study. A statistically significant difference was seen in Ipsilateral Lung Dmean (2.9 Gy *vs*. 4.8 Gy, p < 0.01), Ipsilateral Lung V20 (4.8% *vs*. 8.7%, p < 0.01), and Ipsilateral Lung V8 (10.6% *vs*. 14.5%, p < 0.01) between the 5-fraction group and the 15-fraction group, respectively. In left-sided breast cancer patients, the median value of Heart Dmean dose was significantly lower in the 5-fraction group compared to the 15-fraction group (0.9 Gy *vs*. 2.1 Gy, p < 0.01). Similarly, the 5-fraction group demonstrated lower Heart V8 (0.7% *vs*. 4.1%, p = 0.02), lower median LAD dose (2.3 Gy *vs*. 10.1 Gy, p < 0.01), and maximum LAD dose (10 Gy *vs*. 35.3 Gy, p < 0.01), respectively.

Ratosa *et al*.^20^ compared 3D CRT and volumetric modulated arc therapy (VMAT) in their study of UHF in patients with early breast cancer with or without lymph node irradiation. Treatment planning parameters for OARs in both groups showed: Ipsilateral Lung Dmean of 5.9 Gy (range, 2.2–7.7), Ipsilateral Lung V12 Gy of 16.1% and median mean dose of the heart was 1.3 Gy (range, 0.2–5.6). The median mean heart dose in this study was higher than in our study, but if we take out the VMAT group (that treated patients with internal mammary nodes irradiation) median heart mean dose was 0.7 Gy, similar to our study.

Onsiri *et al*.^21^ publication made dosimetric comparison on OARs in patients with breast cancer treated with photon or proton radiotherapy using UHF regimen and intensity modulated proton therapy (IMPT) or VMAT technique. IMPT showed reduction of the dose to adjacent OARs (Heart Dmean 1.2 Gy relative biological effectiveness (RBE), Ipsilateral Lung Dmean 7.8–8.0 Gy, RBE) compared do VMAT which might translate into the reduction of late toxicities when compared with the photon plan.

The two referenced studies^20,21^ compared two different radiation techniques or/and modalities, rather than different fractionation schedules. We therefore used their reported OARs dosimetric data to compare it to the findings in the UHF group in our study.

The relevance of reducing OAR doses is highlighted by the simulation study by Kiesl *et al*.^22^, which evaluated how different fractionation regimens influence the 30-year risk of secondary malignancies following postoperative breast cancer irradiation. They selected 20 patients with postoperative radiotherapy for early breast cancer and created 3DCRT or VMAT plans for three different fractionation schedules: conventional 50.4 Gy/28 fx, HF 40.05 Gy/15 fx and UHF 26 Gy/5.2 Gy. They evaluated the risk of secondary malignancies for organs in the irradiation field, which was calculated using a mechanistic model developed by Schneider *et al*.^23^ Based on risk modulation, UHF resulted in significantly lower doses to OARs (absolute additional cases of disease per 10,000 patient-years) for lung cancer (42.8% and 31.2%), contralateral breast cancer (39.4% and 25.7%) and soft tissue sarcoma (58.1% and 20.3%) (p < 0.001) compared to conventional and hypofractionation, respectively. Given that both prospective and retrospective studies require extended follow-up, simulation and modeling approaches—such as those employed in this study – provide a practical means of exploring hypotheses that would otherwise be extremely difficult to evaluate in a timely manner.

The dosimetric findings of our study are consistent with those reported by De Rose *et al*.^24^ Their review underscores the importance of strictly adhering to OARs dose limitations to mitigate longterm complications, including cardiac morbidity, pulmonary toxicity, and radiation-induced secondary cancers. This framework provides important context for interpreting recent evidence on UHF. Although UHF regimens employ larger perfraction doses, they frequently yield lower cumulative incidental exposures to surrounding OARs such as the lung and contralateral breast compared with conventional fractionation. These findings suggest that UHF is not only oncologically effective but also feasible from a dosimetric standpoint, as it is generally manageable to maintain compliance with the proposed dose constraints.

Dosimetric differences observed between HF and UHF fractionation schedules in breast cancer radiotherapy in our study and the other studies mentioned in the discussion^19,20,21^ most likely reflect the variations in dose-per-fraction and planning constraints. Higher fraction sizes are associated with greater biological effect necessitating stricter control of dose homogeneity and OARs limits in UHF schedules. This further results in different beam arrangements and modulation compared with hypofractionation. This interpretation is further supported by the statistically higher PTVeval coverage in the UHF cohort, as observed our study. Although technique selection may also contribute, given that UHF protocols commonly incorporate IMRT, VMAT, or DIBH to accommodate modified constraints, this factor is not applicable here, as all patients in our study were treated with 3D-CRT. Overall, these planning and radiobiological considerations explain why fractionation regimen choice leads to measurable dosimetric differences, even when treating the same anatomical region.

The main limitations of our study are its single-center design, the retrospective nature of the control group, and a moderate sample size. In addition, in our study, the evaluation of the estimated dose to the OARs was performed in a freebreathing simulated CT scan. Ideally, the dose to heart should be evaluated for patients treated using alternative treatment positions (lateral decubitus or prone) or with deep inspiration breath hold (DIBH).

To conclude, FAST Forward was a revolutionary clinical study that brought new aspect in radiotherapy treatment of early breast cancer. Shortening the time for radiotherapy to 1 week treatment was groundbreaking for the comfort of the patients. Open access to data and publications about planning, dosimetry and treatment delivery protocols connected with FAST Forward clinical study, during COVID-19 period, facilitated the adaptation of UHF in many centers around the world.^25^ Over the last three decades IMRT, VMAT and DIBH techniques, together with the increasing adoption of moderately HF and UHF treatment schedules as well as the possibility to offer partial breast radiotherapy to a well-defined patient subset have significantly changed radiotherapy for breast cancer patients. Secondary cardiac and pulmonary comorbidities from radiation are uncommon. Because of their low incidence and long latency, the impact of smaller doses on OARs remains uncertain. The present study is among the first to provide dosimetric data for OARs when comparing UFH with HF radiotherapy. Data from studies such as this can support future conclusions that postoperative UFH in patients with early breast cancer may represent an effective, convenient, cost-efficient^26^, and potentially safer treatment option.
